# Supplementary material for: Satellite glial cells promote regenerative growth in sensory neurons
Source: Nat Commun. 2020 Sep 29;11:4891. doi: 10.1038/s41467-020-18642-y (PMC7524726; doi:10.1038/s41467-020-18642-y)
Supplement: Supplementary file 1 — Supplementary Information [file 41467_2020_18642_MOESM1_ESM.pdf]

Supplementary Information

Satellite glial cells promote regenerative growth in sensory neurons

Oshri Avraham[1], Pan-Yue Deng[2], Sara Jones[1], Rejji Kuruvilla[3], Clay F. Semenkovich[4,2], Vitaly A. Klyachko[2] and Valeria Cavalli[1,5,6,*]

[1] Department of Neuroscience, Washington University School of Medicine, St Louis, MO 63110, USA

[2] Department of Cell Biology and Physiology, Washington University School of Medicine, St Louis, MO 63110, USA

[3] Department of Biology, Johns Hopkins University, Baltimore, MD 21218, USA

[4] Division of Endocrinology, Metabolism & Lipid Research, Washington University School of Medicine, St Louis, MO 63110, USA

[5] Center of Regenerative Medicine, Washington University School of Medicine, St. Louis, MO 63110, USA

[6] Hope Center for Neurological Disorders, Washington University School of Medicine, St. Louis, MO 63110, USA

Corresponding author: Valeria Cavalli, cavalli@wustl.edu

Supplementary Figures 1-6

Supplementary Figure 1: Cluster analysis from scRNAseq of mouse DRG, related to Figure 1

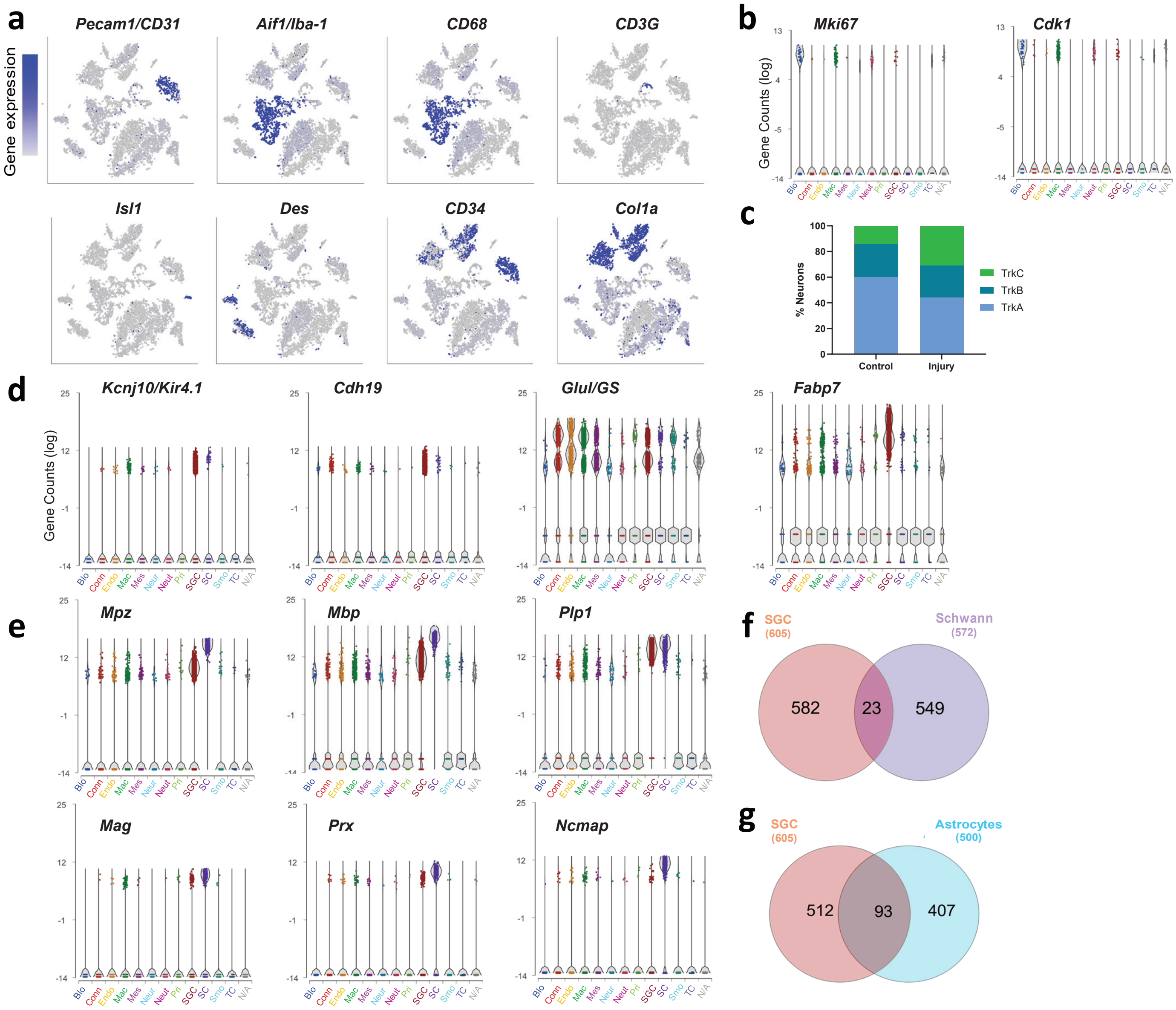

a) t-SNE overlay for expression of marker genes for different cell populations including *Pecam/CD31* for endothelial cells, *Aif1/Iba-1* and *CD68* for Macrophages, *CD3G* for T-cells, *Isl1* for neurons, *Des* for smooth muscle cells, *CD34* for mesenchymal cell and *Col1a1* for connective tissue. The relative levels of expression are presented as a blue color gradient on the left. **b)** Violin plots illustrate the gene counts (log) for *Miki67* and *Cdk1* genes of distinct cell populations after injury. **c)** Fraction of neuronal type within control and injury condition by expression of Trk receptors. n=2 biologically independent experiments. Source data are provided as a Source Data file. **d)** Violin plots illustrate SGC marker genes signatures of distinct cell populations. **e)** Violin plots illustrate Schwann and SGC marker genes signatures in distinct cell populations. **f)** Top differentially expressed genes in the SGC cluster (605 genes) was compared to top differentially expressed genes in the Schwann cell cluster (572 genes). **g)** Top differentially expressed genes in the SGC cluster (605 genes) were compared to the top differentially expressed genes in astrocytes (500 genes).

Supplementary Figure 2: **FABP7 is a specific marker for SGC in adult DRG**, **related to Figure 2**

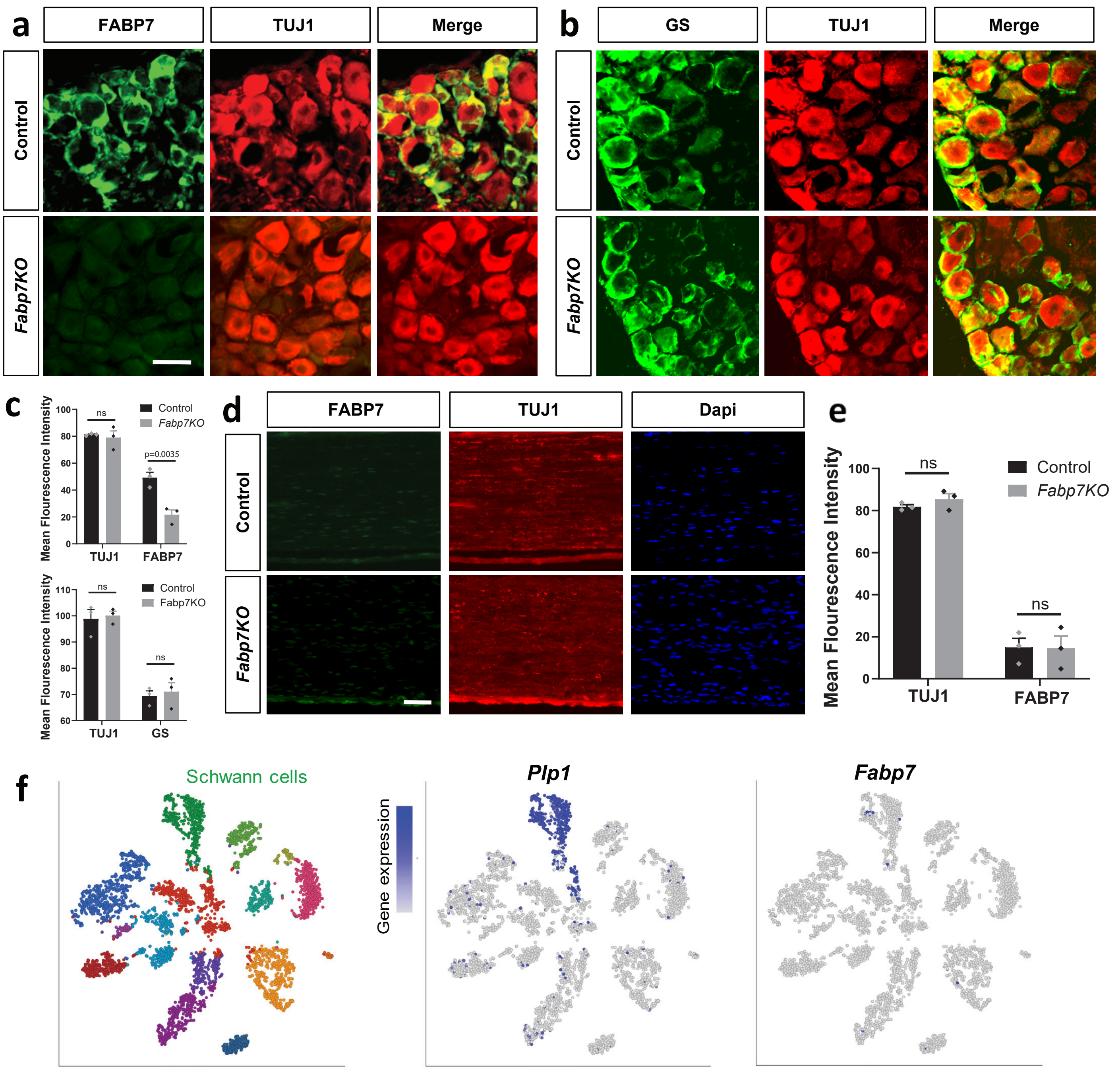

Supplementary Figure 2: **FABP7 is a specific marker for SGC in adult DRG**, **related to Figure 2**
a) DRG sections from *Fabp7KO* and control mice were immunostained for FABP7 (green) and TUJ1 (red). Scale bar: 50 µm. Experiment was repeated independently 3 times. **b)** DRG sections from *Fabp7KO* and control mice were immunostained for Glutamine Synthase (GS) (green) and Tuj1 (red). **c)** Quantification of DRG mean fluorescence intensity of FABP7/GS and TUJ1. **d)** Longitudinal sections of sciatic nerves from Fabp7KO and control mice immunostained for FABP7 (green), TUJ1 (red) and Dapi. Scale bar: 50 µm. **e)** Quantification of nerves mean fluorescence intensity of FABP7 and TUJ1. **f)** t-SNE plot of injured sciatic nerve (9 days post injury) analyzed from the single cell data (Carr et al., 2018). t-SNE overlay for the expression of *Plp1* gene, indicating the Schwann cell cluster, and *Fabp7*. Experiment was repeated independently 3 times in c and e. p-value by Two-way ANOVA in c and e. ns-non significant. Data are presented as mean values±SEM. Source data are provided as a Source Data file for c and e.

Supplementary Figure 3: Pathway analysis of differentially upregulated genes in major cell types, related to Figure 3

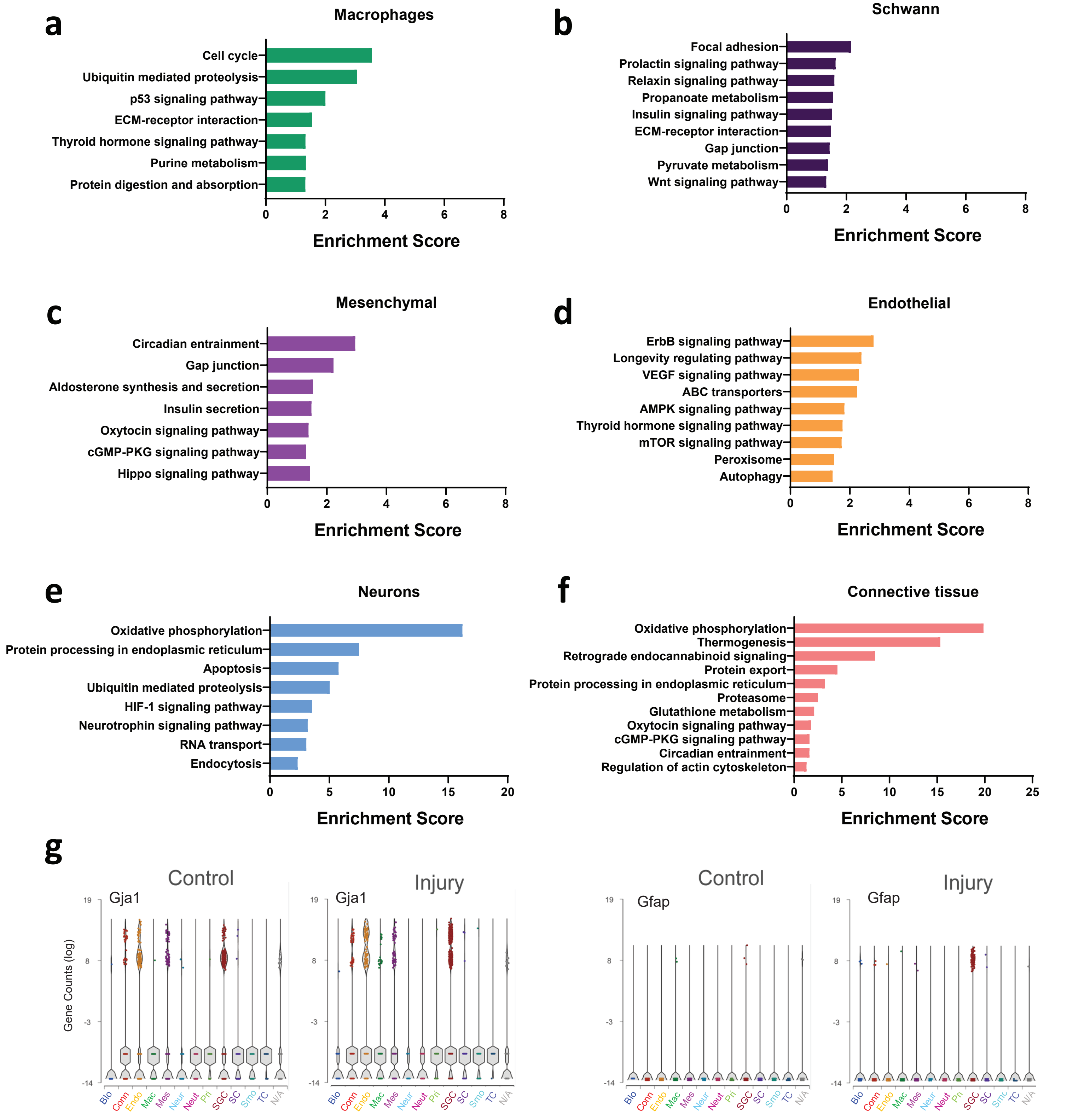

(a-f) Pathway analysis of differentially upregulated genes in major cell types in the DRG following nerve injury (KEGG 2016). **g)** Violin plots illustrate SGC injury induced genes *Gja1* and *Gfap* signatures in distinct cell populations in control and injury conditions.

Supplementary Figure 4: ***Fasn* deletion in SGC does not lead to neuronal cell death or abnormal functional properties, related to Figure 5**

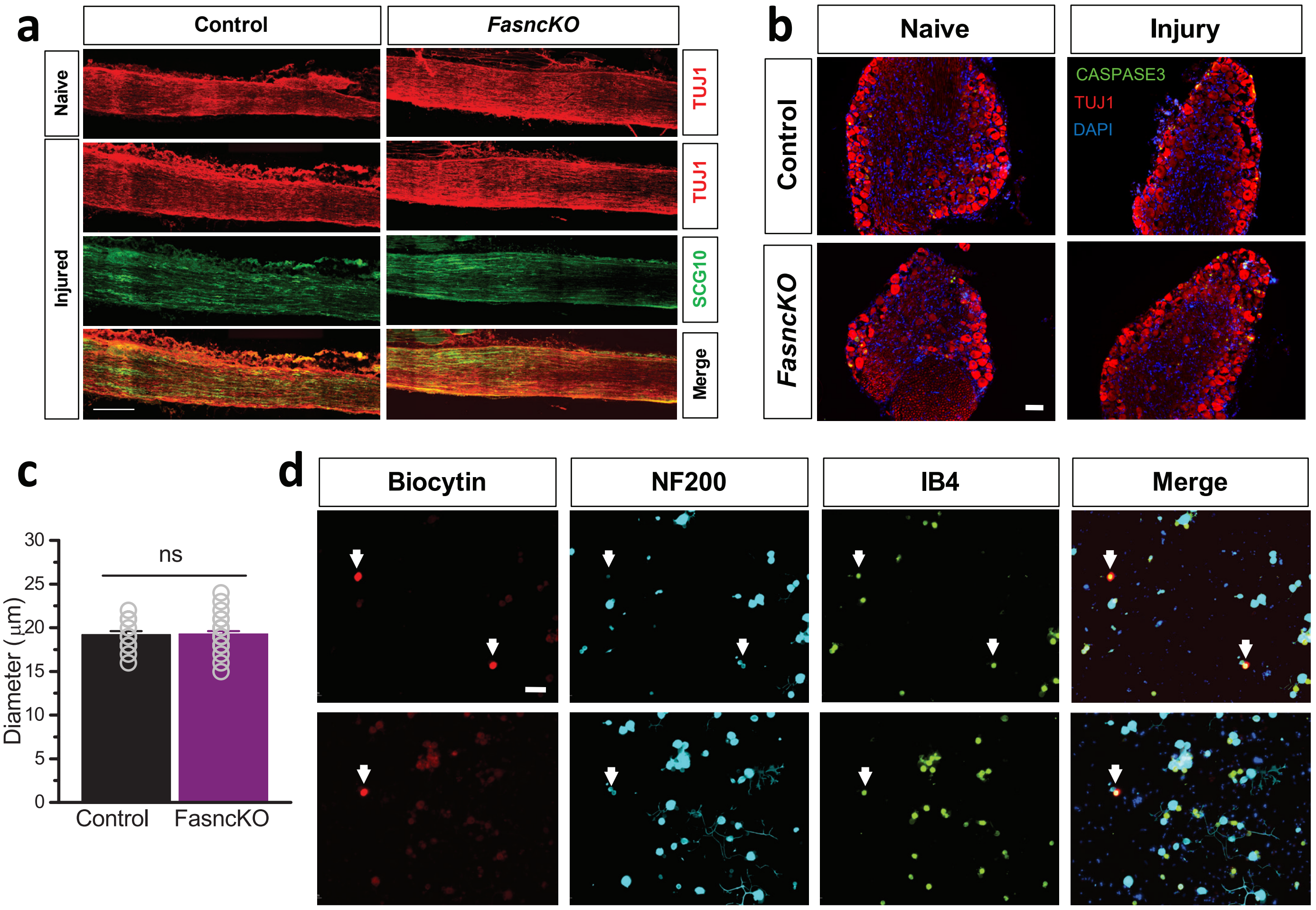

a) Representative images of longitudinal nerve sections from naïve and injured control and *FasncKO* mice, immunostained for TUJ1(red) and SCG10(green). Scale bar: 500 µm. **b)** DRG sections from *FasncKO* and control mice in naïve and injured (3 days post injury) were immuonostained for Cleaved Caspase3 (green), TUJ1 (red) and DAPI (Blue). Scale bar: 50 µm. **c)** Whole-cell recordings in dissociated co-cultures of DRG neurons and glia. Medium diameter neurons (control 19.19 ± 0.42 µm, n = 16 cells; *FasncKO* 19.27 ± 0.34 µm, n = 30 cells, p = 0.88; that were associated with at least one SGC, were targeted for recordings. two tailed t-test ns- non significant. Data are presented as mean values ±SEM. Source data are provided as a Source Data file. **d)** A subset of recorded cells was filled with biocytin (red) via the patch pipette for post hoc verification of neuronal identity; IB4- nociceptors (green) NF200- Proprioceptors/LTMRs (Cyan). Scale bar: 50 µm. Experiments were repeated independently 5 times in a and d and 3 times in b.

Supplementary Figure 5: Activation of PPARα in pure neuronal cultures does not enhance axon regeneration, related to Figure 7

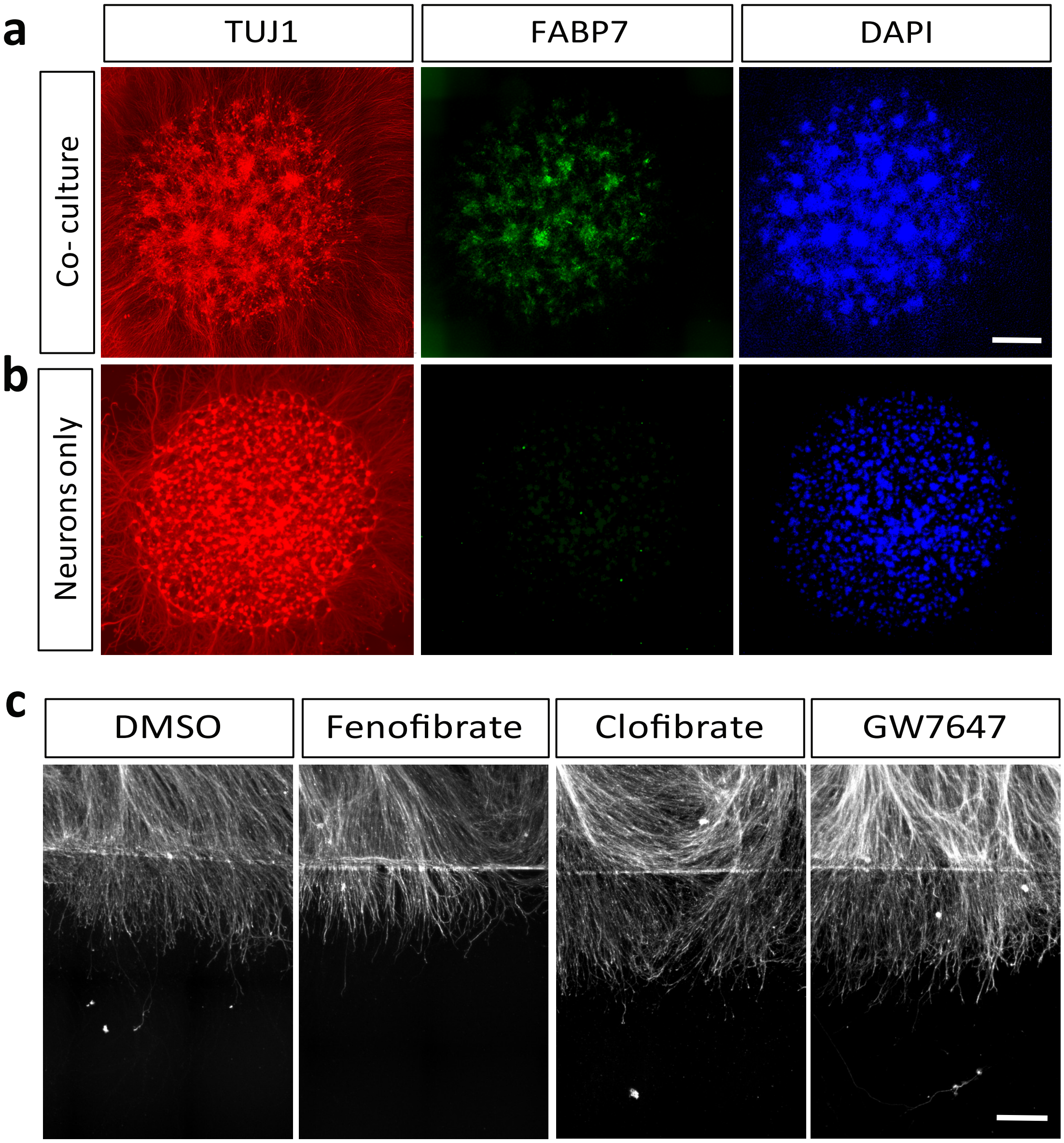

a) Embryonic DRG were dissociated and plated as a spot without 5-fluorodeoxyuridine (FDU) and stained for FABP7 (green), TUJ1 (red) and DAPI (blue) at DIV6. Scale Bar: 250 µm. Experiment was repeated independently 5 times. **b**) Embryonic DRG were dissociated and plated as a spot with 5-fluorodeoxyuridine (FDU). Experiment was repeated independently 5 times. **c)** Embryonic DRG spot co-culture, supplemented with FDU, were axotomized at DIV7 after a 24 h pre-treatment with the indicated PPARα agonists fenofibrate (10µM), Clofibrate (100nM) and GW7647 (10µM). Cultures were fixed after 24h and stained with SCG10. Scale Bar: 250 µm. Experiment was repeated independently 3 times.

Supplementary Figure 6: Neuronal pro-regeneration genes expression in response to fenofibrate, related to figure 7, 8

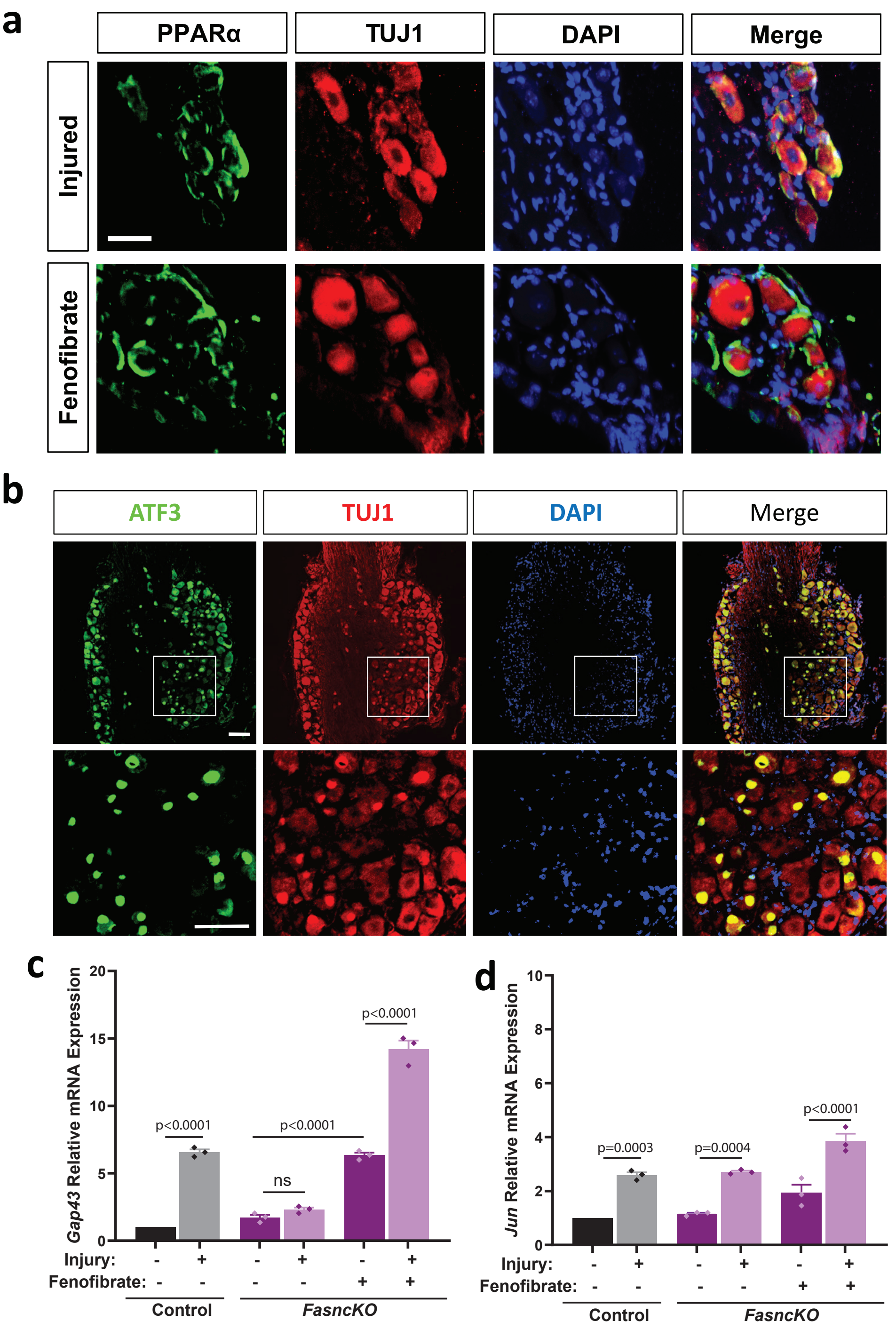

a) Representative images of DRG sections immunostained for PPARα (green) and TUJ1 (red) from mice that received a prior nerve injury or received fenofibrate treatment., Scale bar: 100 µm. **b)** Representative images of injured DRG sections (3 days post injury) immunostained for ATF3 (green), TUJ1(red) and DAPI(Blue) Scale Bar: 100 µm. **c)** qPCR analysis of *Gap43* expression in DRG from control and *FasncKO* mice in naïve and 3 days after sciatic nerve injury with and without fenofibrate. ns-non significant. **d)** qPCR analysis of *Jun* expression in DRG from control and *FasncKO* mice in naïve and 3 days after sciatic nerve injury with and without fenofibrate. n=3 independent animals in a and b. Experiments in c and d was repeated independently 3 times. p-values by One way ANOVA followed by Sidak's multiple comparisons test. Data are presented as mean values ±SEM. Source data are provided as a Source Data file for c and d.
