# Supplementary material for: Satellite glial cells promote regenerative growth in sensory neurons
Source: Nat Commun. 2020 Sep 29;11:4891. doi: 10.1038/s41467-020-18642-y (PMC7524726; doi:10.1038/s41467-020-18642-y)
Supplement: Supplementary file 3 — Description of Additional Supplementary Files [file 41467_2020_18642_MOESM3_ESM.pdf]

Description of Additional Supplementary Files

Title: Supplementary Data 1

Description: Top 10 expressed genes in each cell cluster (related to figure 1c). Calculated expression difference of each gene between a cell cluster and the average in the rest of the clusters (ANOVA fold change threshold >1.5).

Title: Supplementary Data 2

Description: Comparisons of genes expressed in SGC, Schwann cells, astrocytes and myelinating/non myelinating Schwann cells (related to supplementary figure 1f,g). Comparison of the top differentially expressed genes in SGC (605 genes) and Schwann cells (572 genes) (fold-change >4, significant differences across groups by ANOVA, and $p<0.05$ compared to average gene expression in all other populations in the DRG) or astrocytes (500 genes, >6 fold change compared to other populations in the cerebral cortex).

Title: Supplementary Data 3

Description: Differentially expressed genes in SGC after nerve injury (related to figure 3a,7b) Cells in the SGC cluster were pooled, control and injury conditions were compared to identify differentially expressed genes (FDR>0.05, Log2Fold change>2).

Title: Supplementary Data 4

Description: Enriched pathways after injury in SGC with the corresponding genes (related to figure 3a). Differentially expressed genes in SGC analyzed for enriched biological pathways using KEGG 2016 (Kyoto Encyclopedia of Genes and Genomes).
