# Supplementary material for: Satellite glial cells promote regenerative growth in sensory neurons
Source: Nat Commun. 2020 Sep 29;11:4891. doi: 10.1038/s41467-020-18642-y (PMC7524726; doi:10.1038/s41467-020-18642-y)
Supplement: Supplementary file 8 — Reporting Summary [file 41467_2020_18642_MOESM8_ESM.pdf]

nature research

Corresponding author(s): Valeria Cavalli

Last updated by author(s): Aug 31, 2020

Reporting Summary

Nature Research wishes to improve the reproducibility of the work that we publish. This form provides structure for consistency and transparency in reporting. For further information on Nature Research policies, see Authors & Referees and the Editorial Policy Checklist .

Statistics

For all statistical analyses, confirm that the following items are present in the figure legend, table legend, main text, or Methods section.

n/a	Confirmed	
[ ]	[x]	The exact sample size (*n*) for each experimental group/condition, given as a discrete number and unit of measurement
[ ]	[x]	A statement on whether measurements were taken from distinct samples or whether the same sample was measured repeatedly
[ ]	[x]	The statistical test(s) used AND whether they are one- or two-sided<br>*Only common tests should be described solely by name; describe more complex techniques in the Methods section.*
[x]	[ ]	A description of all covariates tested
[ ]	[x]	A description of any assumptions or corrections, such as tests of normality and adjustment for multiple comparisons
[ ]	[x]	A full description of the statistical parameters including central tendency (e.g. means) or other basic estimates (e.g. regression coefficient) AND variation (e.g. standard deviation) or associated estimates of uncertainty (e.g. confidence intervals)
[ ]	[x]	For null hypothesis testing, the test statistic (e.g. *F*, *t*, *r*) with confidence intervals, effect sizes, degrees of freedom and *P* value noted<br>*Give P values as exact values whenever suitable.*
[x]	[ ]	For Bayesian analysis, information on the choice of priors and Markov chain Monte Carlo settings
[x]	[ ]	For hierarchical and complex designs, identification of the appropriate level for tests and full reporting of outcomes
[ ]	[x]	Estimates of effect sizes (e.g. Cohen's *d*, Pearson's *r*), indicating how they were calculated

Our web collection on statistics for biologists contains articles on many of the points above.

Software and code

Policy information about availability of computer code

Data collection	Single-cell RNA-Seq libraries were prepared using GemCode Single-Cell 3′ Gel Bead and Library Kit (10x Genomics)
Data analysis	A digital expression matrix was obtained using 10X's CellRanger pipeline. Quantification and statistical analysis were done with Partek Flow package

For manuscripts utilizing custom algorithms or software that are central to the research but not yet described in published literature, software must be made available to editors/reviewers. We strongly encourage code deposition in a community repository (e.g. GitHub). See the Nature Research guidelines for submitting code & software for further information.

Data

Policy information about availability of data

All manuscripts must include a data availability statement. This statement should provide the following information, where applicable:

- Accession codes, unique identifiers, or web links for publicly available datasets
- A list of figures that have associated raw data
- A description of any restrictions on data availability

Data that support the findings of this study have been deposited in the NCBI GEO database under the accession number GSE139103. (secure token access code for reviewers : wtabciuorlcrhmd). Figures associated raw data: 1,S1,3,S3,6

Field-specific reporting

Please select the one below that is the best fit for your research. If you are not sure, read the appropriate sections before making your selection.

Life sciences study design

All studies must disclose on these points even when the disclosure is negative.

Sample size	No statistical methods were used to predetermine sample sizes, but sample sizes used are similar to sample sizes generally employed in the field for similar experiments.
Data exclusions	There were no data exclusions
Replication	For scRNAseq experiments 6,541 cells from 2 biological replicates were analyzed. Each batch had pooled cells from 5 mice (fig. 1,S1,3,S3,6)<br>For Immunostaining experiments 3 mice were used (Fig.2,S2,3,S4).<br>For transgenic mice with GFP reporter expression, 3 mice (rosaGFP) and 4 mice (Sun1GFP) were used (Fig.2)<br>For western blot analysis, 3 mice for each condition were used (Fig.3)<br>For EM experiments, 3 mice for each genotype were used (Fig.4)<br>For qPCR experiments, RNA was isolated and quantified from3 mice for each condition (Fig.4,7,S6)<br>For whole-cell electrophysiology recording and analysis, 16 cells from 5 control mice and 30 cells from 5 FasncKO mice were used (Fig.4,S4)<br>For in-vivo axon regeneration assay, 8 mice were used for each condition (Fig. 5,7)<br>For ex-vivo axon regeneration assay, 8 mice were used for each condition (Fig.5,7)<br>For embryonic DRG co-culture experiments, 4 biological replicates were used (Fig.6,S5)<br>All experiments were reproducible
Randomization	Animals were randomly chosen for injury or treatments with fenofibrate diet or normal diet.
Blinding	Quantifications were performed by a blinded experimenter to genotype and treatment.

Reporting for specific materials, systems and methods

We require information from authors about some types of materials, experimental systems and methods used in many studies. Here, indicate whether each material, system or method listed is relevant to your study. If you are not sure if a list item applies to your research, read the appropriate section before selecting a response.

Materials & experimental systems

n/a	Involved in the study	
☐	☒	Antibodies
☒	☐	Eukaryotic cell lines
☒	☐	Palaeontology
☐	☒	Animals and other organisms
☒	☐	Human research participants
☒	☐	Clinical data

Methods

n/a	Involved in the study	
☒	☐	ChIP-seq
☒	☐	Flow cytometry
☒	☐	MRI-based neuroimaging

Antibodies

Antibodies used	SCG10/Stmn2 (1:1000; Novus catalog #NBP1-49461, RRID:AB_10011569),<br>Tubb3/bIII tubulin antibody (BioLegend catalog #802001, RRID:AB_291637), Griffonia<br>simplicifolia isolectin B4 (IB4) directly conjugated to Alexa Fluor 488 or Alexa Fluor 594 (Thermo<br>Fisher Scientific catalog #I21411 and #I21413), Fabp7 (Thermo Fisher Scientific Cat# PA5-<br>24949, RRID:AB_2542449), cleaved caspase 3 (CST Cat# 9664, RRID:AB_2070042), Fasn<br>(Abcam, Catalog #ab128870), Glutamine synthase (Abcam, Catalog #ab49873), Gapdh (Santa Cruz, catalog# sc25778), Pparα (Thermo Fisher Scientific, Catalog # PA1-822A).
Validation	SCG10/Stmn2 -Genetic Strategy Validation- Expression of the target protein is compared before and after knockout or knockdown using CRISPR/CAS9 or siRNA/shRNA. If protein expression following knockout or knockdown is substantially reduced, then antibody specificity is ensured (manufacturer's website).<br>Tubb3/bIII -validated for WB, reported in the literature for IF (Flores-Otero J, et al. 2007. J. Neurosci. 27:14023, Tseung G, et al. 2011. J. Virol. 85:5718).<br>IB4- reported in the literature for IF (A sensory-labeled line for cold: TRPM8-expressing sensory neurons define the cellular basis for cold, cold pain, and cooling-mediated analgesia. Knowlton WM, Palkar R, Lippoldt EK, McCoy DD, Baluch F, Chen J, McKemy DD. J Neurosci (2013) 33:2837-2848)<br>Fabp7- Validation for IF provided in the manuscript using Fabp7KO mice (fig. S2).<br>cleaved caspase 3- Validation for IF provided in the literature

Fasn- Validated using positive controls in HeLa, 293T and A549 cell lysates; Human liver tissue; A549 cells (from manufacturer's website).
Glutamine synthase- Validated using positive controls in Rat brain tissue, rat brain cytosolic fraction extract, cerebellum, kidney (from manufacturer's website).
Gapdh- Validation for WB provided in the literature
Pparα- Validation for target specificity and functional application provided in the manufacturer's website

Animals and other organisms

Policy information about studies involving animals; ARRIVE guidelines recommended for reporting animal research

Laboratory animals	C57Bl/6 or the indicated genotype 8-12 weeks old week old, were used for all experiments. C57Bl/6 Females only were used for scRNAseq experiments. For all other experiments male and female were used.
Wild animals	The study did not involve wild animals
Field-collected samples	The study did not involve samples collected from the field
Ethics oversight	All surgical procedures were approved by Washington University in St. Louis School of Medicine Institutional Animal Care and Use Committee’s regulations.

Note that full information on the approval of the study protocol must also be provided in the manuscript.
